# Impact of Sleeve Gastrectomy Versus Intensive Lifestyle Modifications With Obesity Management Medications on BMI Trajectory and Target Attainment: A Prospective Matched Cohort Study

**DOI:** 10.1111/dom.70912

**Published:** 2026-05-31

**Authors:** Maria Francesca Russo, Giovanni Casella, Giulia Angelini, Sara Russo, James Casella‐Mariolo, Vito D'Andrea, Giulio Illuminati, Lidia Castagneto‐Gissey

**Affiliations:** ^1^ Department of Surgery Sapienza University of Rome Rome Italy; ^2^ Department of Medical and Surgical Sciences Fondazione Policlinico Universitario A. Gemelli IRCCS Rome Italy; ^3^ Department of General and Emergency Surgery Ospedale Dei Castelli (NOC) Rome Italy

**Keywords:** antiobesity drug, bariatric surgery, effectiveness, weight management

## Abstract

**Aims:**

To compare sleeve gastrectomy (SG) with intensive lifestyle modification combined with obesity management medications (ILM/OMMs) in adults with severe obesity, focusing on BMI trajectory, time to achieving clinically meaningful weight loss, and metabolic outcomes in a real‐world clinical setting.

**Materials and Methods:**

This prospective matched cohort study included 190 patients (BMI ≥ 35 kg/m^2^, obesity‐related comorbidities) assigned to SG or ILM/OMM in a 1:1 ratio, matched for age, sex, BMI, and diabetes duration. The primary outcomes were change in BMI at 12 months and time to achieving ≥ 25% total weight loss (TWL). Secondary outcomes included changes in weight and obesity‐related comorbidities (hypertension, dyslipidemia, type 2 diabetes) (clinicalTrials.gov, NCT06820047).

**Results:**

At 12 months, model‐based BMI was lower after SG than after ILM/OMM (28.4 kg/m^2^, 95% CI 27.2 to 29.6 vs. 31.6 kg/m^2^, 95% CI 30.5 to 32.8; between‐group difference −3.2 kg/m^2^, 95% CI −4.9 to −1.5; *p* < 0.001). Estimated %TWL was greater after SG than after ILM/OMM at 12 months (37.6%, 95% CI 36.5 to 38.7 vs. 18.0%, 95% CI 16.9 to 19.1; difference 19.6 percentage points, 95% CI 18.1 to 21.1; *p* < 0.001). Time to achieving ≥ 25% TWL was shorter after SG; the median time was 6 months in the SG group, whereas the median was not reached by 12 months in the ILM/OMM group (log‐rank *p* < 0.001; HR 13.85, 95% CI 7.81–24.58). Both groups showed metabolic improvement, although between‐group differences were limited.

**Conclusions:**

SG was associated with greater and more rapid weight loss than ILM/OMM over 1 year, with earlier achievement of ≥ 25% TWL. Both approaches produced clinically meaningful metabolic improvements, supporting individualized treatment selection.

## 1 Introduction

Metabolic and bariatric surgery is currently the most effective intervention for achieving substantial and sustained weight loss in individuals with severe obesity. Surgical procedures are recommended for individuals with a body mass index (BMI) ≥ 35 kg/m^2^ regardless of the presence of obesity‐related comorbidities [1]. In addition to producing marked weight reduction, metabolic surgery has demonstrated substantial metabolic benefits, including improved glycemic control and higher rates of type 2 diabetes remission compared with conventional medical management [2, 3, 4].

Although lifestyle interventions remain the cornerstone of obesity management, their long‐term effectiveness is often limited, particularly in individuals with severe obesity [5]. The landscape of pharmacological treatment of obesity has evolved rapidly with the development of incretin‐based therapies. Glucagon‐like peptide‐1 receptor agonists (GLP‐1RAs), such as semaglutide, promote weight loss through appetite suppression, delayed gastric emptying, and increased satiety [6, 7, 8]. Randomized clinical trials have shown that semaglutide can produce average weight reductions of approximately 15% of baseline body weight, substantially greater than those achieved with lifestyle intervention alone [8]. These advances have expanded the range of non‐surgical treatment options available for patients with severe obesity.

Despite these developments, direct comparisons between metabolic surgery and intensive medical therapy remain limited, particularly in real‐world clinical populations. Most studies focus on mean weight loss outcomes at fixed time points, whereas less attention has been given to the timing and likelihood of achieving clinically meaningful weight‐loss thresholds. Understanding not only the magnitude of weight loss but also how quickly patients reach substantial weight‐loss targets may be important for clinical decision‐making and patient counselling.

This study seeks to extend current evidence by incorporating trajectory‐based assessment of weight loss and time to clinically meaningful thresholds within a real‐world matched cohort with complete follow‐up. Therefore, the aim of the present prospective matched cohort study was to compare sleeve gastrectomy (SG) with intensive lifestyle modification combined with obesity management medications (ILM/OMMs) in adults with severe obesity in a real‐world clinical setting. Specifically, differences in BMI trajectories, the time required to achieve ≥ 25% total weight loss, and changes in obesity‐related metabolic comorbidities over 1 year of follow‐up were evaluated.

## 2 Materials and Methods

### 2.1 Study Design

This study is a 1‐year multicenter, matched, two‐arm, prospective study comparing the efficacy of ILM/OMMs to bariatric surgery, specifically SG.

**FIGURE 1:**
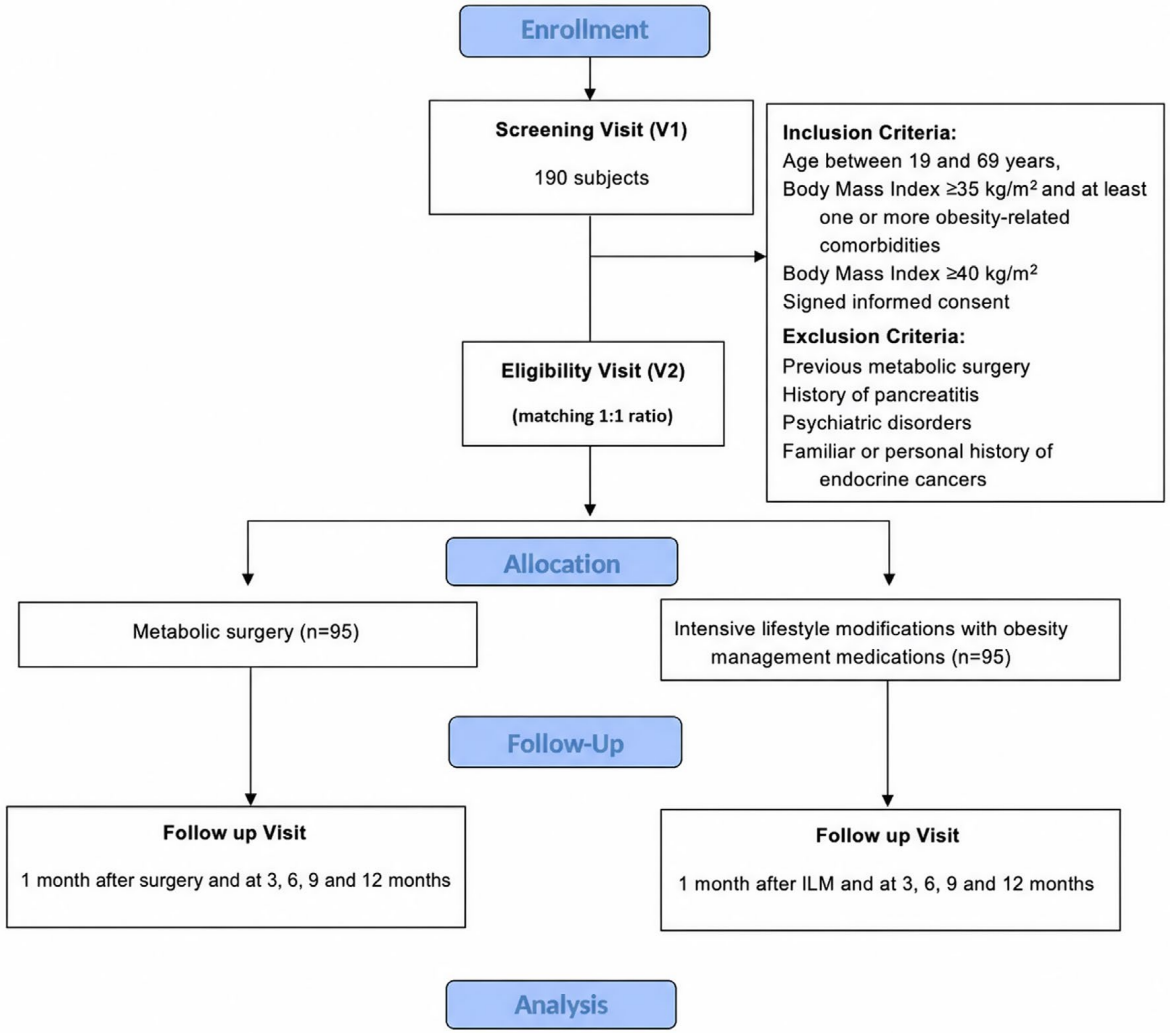
Study flow chart.

Patients attending the weight‐loss clinic of Sapienza University Hospital and Catholic University Hospital in Rome, Italy, and eligible for bariatric surgery were allocated to intensive lifestyle modifications with obesity management medications or metabolic surgery. Ninety‐five patients per arm were matched in a 1:1 ratio, pairing one patient who underwent SG with one treated with ILM/OMMs. The matching process accounted for the following variables: sex, age, BMI, and diabetes duration (measured from diagnosis). Study flow chart is reported in Figure 1. The study was conducted between May 2022 and July 2024. This study followed the STROBE (STrengthening the Reporting of OBservational studies in Epidemiology) reporting guidelines for cohort studies.

The trial was approved by the ethics committee of both institutions and conducted in accordance with the principles of the Declaration of Helsinki and Good Clinical Practice guidelines. All patients provided their written informed consent before participation, and an additional written informed consent was signed before bariatric surgery.

This study is registered at ClinicalTrials.gov (NCT06820047).

### 2.2 Participants

Inclusion criteria were age between 19 and 69 years with stable body weight in the past 6 months; BMI ≥ 35 kg/m^2^ and at least one or more obesity‐related co‐morbidities (T2D, hypertension, sleep apnea and other respiratory disorders, metabolic‐associated fatty liver disease, osteoarthritis, lipid abnormalities, gastrointestinal disorders, or heart disease) or BMI ≥ 40 kg/m^2^. Exclusion criteria were previous bariatric surgery; history of pancreatitis, severe psychiatric disorders; and personal or familial history of endocrine cancers.

### 2.3 Interventions

Treatment allocation reflected routine clinical practice and was determined through a shared decision‐making process between the multidisciplinary obesity team and the patient. Factors considered included patient preference, surgical eligibility according to current international guidelines, comorbidity profile, willingness to undergo surgery, and contraindications to pharmacological treatment.

#### 2.3.1 Medical Arm

Patients underwent 1 month of a very low calorie diet (VLCD) with 813 kcal/day, followed by a structured low calorie diet (LCD) with 1200 kcal/day for 11 months together with intensive physical exercise (30 min/day of brisk walking plus at least 3 h/week of aerobic exercise). Pharmacological treatment primarily consisted of semaglutide (Ozempic), given once weekly as subcutaneous injections starting at 0.25 mg and titrated every 4 weeks to 0.5 mg, 1.0 mg, and up to 2.4 mg as tolerated, in accordance with routine clinical practice. Patients were evaluated at baseline and at 1, 3, 6, 9, and 12 months after starting the interventions. The program was delivered within a multidisciplinary framework involving physicians and dietitians, with regular follow‐up visits throughout the study period.

#### 2.3.2 Surgical Arm

Patients underwent SG, involving longitudinal resection of approximately 75% of the stomach along its greater curvature, with excision of the fundus and part of the body and antrum, preserving a portion of the latter and the pylorus itself. This results in a vertical tube‐shaped gastric tube “sleeve”. After surgery, patients followed a free diet. Patients were evaluated at baseline and at 1, 3, 6, 9, and 12 months after starting the intervention.

### 2.4 Outcome Measures

The primary endpoint was the change in BMI at 1 year and time to achieve a 25% reduction in total weight loss (TWL). A 25% TWL threshold was selected as a clinically meaningful benchmark commonly achieved after bariatric surgery. Although standardized criteria for surgical success exist, including definitions based on excess weight loss as proposed by ASMBS/IFSO guidelines [1], the ≥ 25% TWL threshold was selected as a pragmatic comparative benchmark rather than a formal definition of treatment success. Secondary endpoints were changes at 1 year from baseline in weight, plasma glucose, lipid profile, and comorbidities, namely hypertension, CVD, dyslipidemia, T2D.

Time to achievement of ≥ 25% TWL was evaluated using time‐to‐event methods. The event was defined as the first follow‐up visit at which TWL reached or exceeded 25%. Follow‐up time was measured from baseline to the visit when the threshold was first achieved (1, 3, 6, 9, or 12 months). Participants who did not reach the threshold during follow‐up were censored at the last available visit. Kaplan–Meier curves were constructed to estimate the cumulative probability of achieving ≥ 25% TWL according to treatment strategy. Differences between groups were assessed using the log‐rank test. Cox proportional hazards regression was used to estimate the hazard ratio (HR) and corresponding 95% confidence interval (CI) for achieving ≥ 25% TWL in the SG group compared with the intensive lifestyle modification plus pharmacotherapy group.

Body weight and height were measured using calibrated scales and with subjects wearing light clothing. Percentage of total body weight loss was calculated using the formula: (starting weight minus current weight)/(starting weight) × 100.

### 2.5 Sample Size

Sample size was calculated assuming the non‐inferiority of Semaglutide 2.4 mg plus ILM with respect to SG. For establishment of equivalence between surgical group and low‐calorie diet group, under the hypothesis of a true mean difference of 3% (weight loss of 25% in the low‐calorie diet group versus 28% in the surgical group) and of an equivalence limit of 5%, with a standard deviation of 3.4%, the sample size required is 76 patients per group (α = 0.05 and a power of 90%). If a 20% retention rate is considered, then the total number of patients to be included in the study will be 190 (95 per group).

The study flow chart is illustrated in Figure 1.

### 2.6 Statistical Analysis

High responders were defined as patients achieving weight loss at or above the 75th percentile of the overall cohort (≥ 32.7% TWL), while low responders were those at or below the 25th percentile (≤ 13.0% TWL). Logistic regression models were constructed to identify baseline characteristics associated with high and low response, including demographic factors, anthropometric measurements, and comorbidities.

Continuous longitudinal outcomes, including body weight, BMI and %TWL, were analysed using linear mixed‐effects models including fixed effects for treatment group, visit and treatment‐by‐visit interaction, with a patient‐level random intercept to account for within‐subject correlation. Estimated marginal means and between‐group contrasts were reported with 95% confidence intervals at each follow‐up visit. Model diagnostics and fit indices for the continuous mixed‐effects models are reported in Table S3. Repeated binary outcomes, including hypertension, cardiovascular disease, dyslipidemia, type 2 diabetes and diabetes medication use, were analysed using mixed‐effects logistic regression with fixed effects for treatment group, visit and treatment‐by‐visit interaction and a patient‐level random intercept. Observed *n* (%) values were retained for transparency, and model‐based predicted probabilities and between‐group differences were reported with 95% confidence intervals.

Time to achievement of ≥ 25% TWL was analysed using Kaplan–Meier curves, the log‐rank test, and Cox proportional hazards regression. To address potential residual confounding, we performed a propensity score sensitivity analysis using baseline covariates only, excluding obesity medication because it was initiated after enrollment and therefore was not a baseline confounder.

Baseline comparisons were performed using Student's *t*‐test and Fisher's exact test, whereas longitudinal outcomes were analysed using mixed‐effects models. A *p* < 0.05 was considered statistically significant. All analyses were performed using R version 4.2.0 [9] and replicated for the revision using Python/statsmodels.

## 3 Results

One‐hundred‐ninety patients were assigned to undergo either metabolic surgery, namely SG, or intensive lifestyle monitoring with obesity management medications. Patients were matched 1:1 according to sex, age, BMI, and diabetes duration. Characteristics of patients at baseline are reported in Table 1.

**TABLE 1:** Baseline characteristics of patients undergoing sleeve gastrectomy or intensive lifestyle modification with pharmacotherapy.

	Sleeve gastrectomy (*n* = 95)	ILM (*n* = 95)	*p*
Age, years ± SD	42.5 ± 4.2	48.8 ± 2.1	0.03
Sex, male	29 (30.5%)	16 (16.8%)	0.027
Weight, kg ± SD	124.2 ± 23.1	105.7 ± 18.4	< 0.001
BMI, kg/m^2^ ± SD	45.6 ± 8.1	45.3 ± 8	0.777
Hypertension, *n* (%)	32 (33.7%)	24 (25.3%)	0.204
CVD, *n* (%)	1 (1.1%)	7 (7.4%)	0.031
Dyslipidemia, *n* (%)	17 (17.9%)	6 (6.2%)	0.015
T2D, *n* (%)	16 (16.8%)	12 (12.6%)	0.413
Diabetes medications, *n* (%)	17 (17.9%)	9 (9.5%)	0.092
Metformin, *n* (%)	16 (16.8%)	8 (8.4%)	0.81
GLP1‐RA, *n* (%)	0	6 (6.3%)	0.013
SGLT2‐I, *n* (%)	0	1 (1.1%)	0.317
Insulin, *n* (%)	6 (6.3%)	1 (1.1%)	0.055

*Note:* Values are expressed as mean ± standard deviation or number (percentage).

Abbreviations: BMI, body mass index; CVD, cardiovascular disease; GLP1‐RA, glucagon‐like peptide‐1 receptor agonist; SGLT2‐I, sodium‐glucose cotransporter‐2 inhibitor; T2D, type 2 diabetes.

**FIGURE 2:**
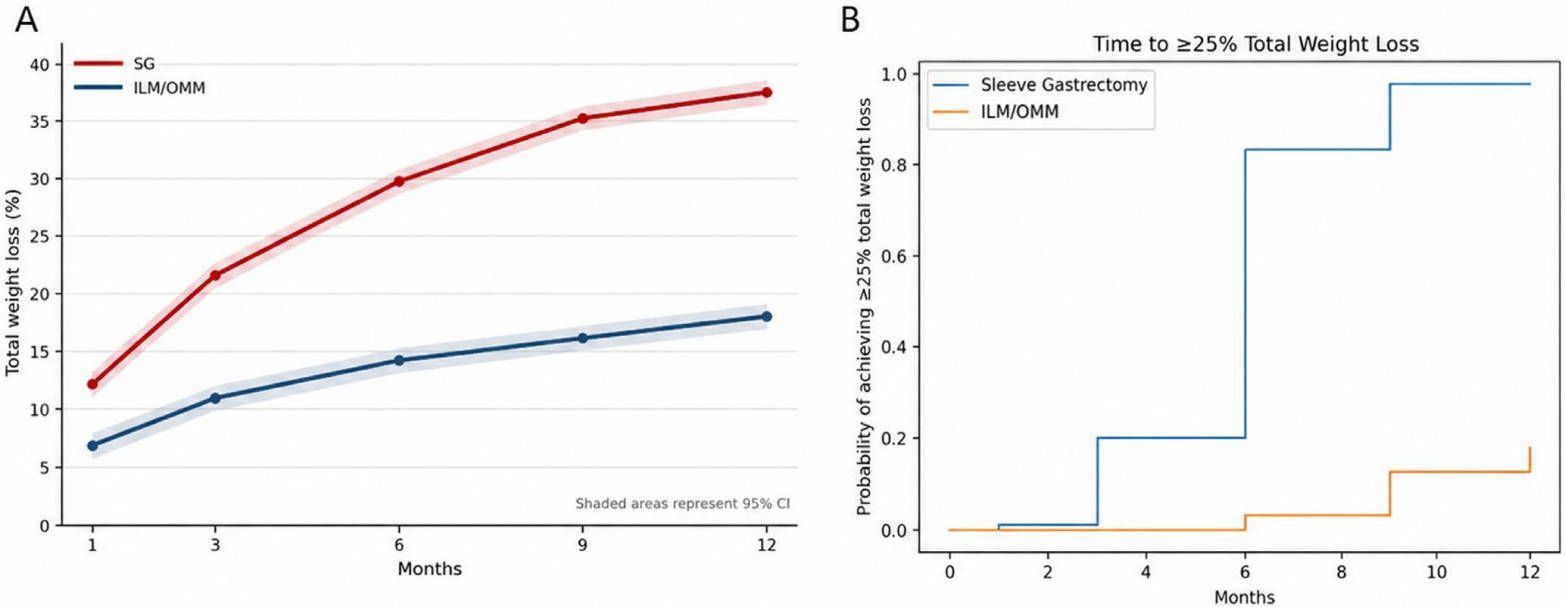
(A) Longitudinal trajectory of %TWL according to treatment group. Points represent estimated mean %TWL at 1, 3, 6, 9 and 12 months, and shaded areas represent 95% confidence intervals. (B) Time to achieving ≥ 25% total weight loss during follow‐up according to treatment strategy.

Patients were followed at 1,3,6,9 and 12 months. No participant was lost to clinical follow‐up; denominators vary for selected repeated binary variables because of incomplete availability of specific medication/comorbidity data at some visits. Percentage of total body weight loss at follow‐up was calculated. Changes are reported in Figure 2A.

### 3.1 Propensity‐Adjusted Analysis

To address potential residual confounding related to the observational study design, a propensity score analysis was performed incorporating baseline age, sex, BMI, body weight, diabetes status, hypertension, and dyslipidemia.

Propensity scores demonstrated adequate overlap between treatment groups, suggesting comparability of the cohorts. After adjustment for the propensity score and baseline covariates, SG remained independently associated with greater weight reduction at 12 months compared with intensive lifestyle modification combined with pharmacotherapy, confirming the robustness of the primary findings (Table S1).

### 3.2 Anthropometric Outcomes

#### 3.2.1 Primary Outcome

At T12, BMI was lower in the SG group than in the ILM/OMM group. In the linear mixed‐effects model, the estimated BMI was 28.4 kg/m^2^ in the SG group (95% CI 27.2–29.6) and 31.6 kg/m^2^ in the ILM/OMM group (95% CI 30.5–32.8). The model‐based between‐group difference was −3.2 kg/m^2^ (95% CI −4.9 to −1.5; *p* < 0.001). The BMI group‐by‐visit interaction was significant (Wald *χ* ^2^ = 178.59, df = 5, *p* < 0.001) (Table 2).

**TABLE 2:** Longitudinal changes in anthropometric parameters and obesity‐related comorbidities over 12 months according to treatment group.

Outcome	Visit	SG estimate (95% CI)	ILM/OMM estimate (95% CI)	Difference (95% CI)	*p*
Weight (kg)	T1	109.1 (105.6 to 112.6)	98.5 (95.0 to 102.0)	10.6 (5.6 to 15.6)	< 0.001
	T3	97.3 (93.8 to 100.9)	94.2 (90.6 to 97.7)	3.2 (−1.8 to 8.1)	0.21
	T6	87.3 (83.8 to 90.8)	90.5 (87.0 to 94.1)	−3.3 (−8.2 to 1.7)	0.198
	T9	80.3 (76.8 to 83.8)	88.3 (84.8 to 91.8)	−8.0 (−12.9 to −3.0)	0.002
	T12	77.2 (73.6 to 80.7)	86.2 (82.7 to 89.7)	−9.0 (−14.0 to −4.0)	< 0.001
BMI (kg/m^2^)	T1	40.1 (38.9 to 41.3)	36.1 (34.9 to 37.3)	4.1 (2.4 to 5.7)	< 0.001
	T3	35.8 (34.6 to 37.0)	34.5 (33.3 to 35.6)	1.4 (−0.3 to 3.0)	0.114
	T6	32.1 (30.9 to 33.3)	33.1 (31.9 to 34.3)	−1.0 (−2.7 to 0.6)	0.226
	T9	29.5 (28.3 to 30.7)	32.4 (31.2 to 33.6)	−2.9 (−4.6 to −1.2)	< 0.001
	T12	28.4 (27.2 to 29.6)	31.6 (30.5 to 32.8)	−3.2 (−4.9 to −1.5)	< 0.001
%TWL	T1	12.1 (11.1 to 13.2)	6.8 (5.8 to 7.9)	5.3 (3.8 to 6.8)	< 0.001
	T3	21.6 (20.5 to 22.6)	10.9 (9.9 to 12.0)	10.6 (9.1 to 12.1)	< 0.001
	T6	29.8 (28.7 to 30.8)	14.2 (13.1 to 15.3)	15.5 (14.0 to 17.1)	< 0.001
	T9	35.3 (34.2 to 36.4)	16.1 (15.1 to 17.2)	19.2 (17.7 to 20.7)	< 0.001
	T12	37.6 (36.5 to 38.7)	18.0 (16.9 to 19.1)	19.6 (18.1 to 21.1)	< 0.001

Outcome	Visit	SG observed *n*/*N* (%)	ILM/OMM observed *n*/*N* (%)	Predicted difference, pp (95% CI)
T2D	T1	10/95 (10.5)	12/95 (12.6)	−2.1 (−11.2 to 7.0)
	T3	10/95 (10.5)	12/95 (12.6)	−2.1 (−11.2 to 7.0)
	T6	9/95 (9.5)	12/92 (13.0)	−3.2 (−12.1 to 5.7)
	T9	8/95 (8.4)	12/95 (12.6)	−4.2 (−12.9 to 4.5)
	T12	7/95 (7.4)	12/92 (13.0)	−5.3 (−13.8 to 3.2)
Diabetes medications	T1	10/95 (10.5)	9/95 (9.5)	1.1 (−7.5 to 9.6)
	T3	8/95 (8.4)	9/95 (9.5)	−1.1 (−9.2 to 7.1)
	T6	7/95 (7.4)	10/92 (10.9)	−3.2 (−11.3 to 4.9)
	T9	6/95 (6.3)	10/95 (10.5)	−4.2 (−12.1 to 3.7)
	T12	5/95 (5.3)	8/91 (8.8)	−4.2 (−11.6 to 3.2)
Metformin	T1	9/95 (9.5)	8/95 (8.4)	1.1 (−7.1 to 9.2)
	T3	8/95 (8.4)	8/95 (8.4)	0.0 (−7.9 to 7.9)
	T6	7/95 (7.4)	8/92 (8.7)	−1.1 (−8.7 to 6.6)
	T9	6/95 (6.3)	8/95 (8.4)	−2.1 (−9.5 to 5.3)
	T12	5/95 (5.3)	6/91 (6.6)	−2.0 (−8.9 to 4.9)
GLP1‐RA	T1	0/95 (0.0)	6/95 (6.3)	−6.3 (−11.2 to −1.4)
	T3	0/95 (0.0)	6/95 (6.3)	−6.3 (−11.2 to −1.4)
	T6	0/95 (0.0)	6/92 (6.5)	−6.3 (−11.2 to −1.4)
	T9	0/95 (0.0)	6/95 (6.3)	−6.3 (−11.2 to −1.4)
	T12	0/95 (0.0)	6/91 (6.6)	−6.3 (−11.2 to −1.4)
SGLT2‐I	T1	0/95 (0.0)	1/95 (1.1)	−1.1 (−3.1 to 1.0)
	T3	0/95 (0.0)	1/95 (1.1)	−1.1 (−3.1 to 1.0)
	T6	0/95 (0.0)	3/92 (3.3)	−3.2 (−6.8 to 0.4)
	T9	0/95 (0.0)	2/95 (2.1)	−2.1 (−5.0 to 0.8)
	T12	0/95 (0.0)	1/91 (1.1)	−1.1 (−3.2 to 1.1)
Insulin	T1	5/95 (5.3)	1/95 (1.1)	4.2 (−0.7 to 9.1)
	T3	4/95 (4.2)	1/95 (1.1)	3.2 (−1.4 to 7.7)
	T6	4/95 (4.2)	1/92 (1.1)	3.2 (−1.4 to 7.7)
	T9	4/95 (4.2)	1/95 (1.1)	3.2 (−1.4 to 7.7)
	T12	4/95 (4.2)	1/91 (1.1)	3.2 (−1.4 to 7.7)
Hypertension	T1	28/95 (29.5)	24/95 (25.3)	4.2 (−8.5 to 16.9)
	T3	22/95 (23.2)	24/95 (25.3)	−2.1 (−14.3 to 10.1)
	T6	19/95 (20.0)	25/92 (27.2)	−6.4 (−18.3 to 5.6)
	T9	18/95 (18.9)	25/95 (26.3)	−7.4 (−19.2 to 4.5)
	T12	15/95 (15.8)	22/91 (24.2)	−9.4 (−20.8 to 2.0)
CVD	T1	1/95 (1.1)	7/95 (7.4)	−6.3 (−12.0 to −0.7)
	T3	1/95 (1.1)	7/95 (7.4)	−6.3 (−12.0 to −0.7)
	T6	1/95 (1.1)	6/92 (6.5)	−6.3 (−11.9 to −0.7)
	T9	1/95 (1.1)	7/95 (7.4)	−6.3 (−12.0 to −0.7)
	T12	1/95 (1.1)	6/91 (6.6)	−6.3 (−12.0 to −0.7)
Dyslipidemia	T1	15/95 (15.8)	6/95 (6.3)	9.5 (0.7 to 18.3)
	T3	15/95 (15.8)	5/95 (5.3)	10.5 (1.9 to 19.1)
	T6	15/95 (15.8)	5/92 (5.4)	10.5 (1.9 to 19.1)
	T9	15/95 (15.8)	5/95 (5.3)	10.5 (1.9 to 19.1)
	T12	14/95 (14.7)	4/91 (4.4)	10.6 (2.3 to 18.8)

*Note:* Continuous outcomes are presented as model‐based estimated marginal means (95% CI) derived from linear mixed‐effects models including treatment group, visit, and treatment‐by‐visit interaction with patient‐level random intercepts. Binary outcomes are presented as observed n/N (%) and model‐based predicted probability differences (95% CI) from mixed‐effects logistic regression models with patient‐level random intercepts. Differences are expressed as SG minus ILM/OMM. Time points correspond to 1, 3, 6, 9, and 12 months.

Abbreviations: BMI, body mass index; CI, confidence interval; CVD, cardiovascular disease; GLP1‐RA, glucagon‐like peptide‐1 receptor agonist; SGLT2‐I, sodium‐glucose cotransporter‐2 inhibitor; T2D, type 2 diabetes.

#### 3.2.2 Time to Achieve 25% Total Weight Loss

Kaplan–Meier analysis demonstrated a shorter time to achieving ≥ 25% total weight loss in patients undergoing SG compared with those receiving ILM/OMMs (Figure 2B). The median time to reach the ≥ 25% TWL threshold was 6 months in the SG group, whereas the median was not reached by 12 months in the ILM/OMM group. The difference between groups was significant according to the log‐rank test (*p* < 0.001). In Cox proportional hazards analysis, SG was associated with a higher probability of achieving ≥ 25% TWL during follow‐up compared with ILM/OMM (HR 13.85, 95% CI 7.81–24.58; *p* < 0.001).

### 3.3 Secondary Outcomes

#### 3.3.1 Weight Changes

Longitudinal mixed‐effects modelling showed greater %TWL after SG than after ILM/OMM at every post‐baseline visit (Table 2 and Figure 2A). The estimated %TWL values were T1: SG 12.1% (95% CI 11.1–13.2) versus ILM/OMM 6.8% (95% CI 5.8–7.9), difference 5.3 percentage points (95% CI 3.8–6.8); T3: SG 21.6% (95% CI 20.5–22.6) versus ILM/OMM 10.9% (95% CI 9.9–12.0), difference 10.6 percentage points (95% CI 9.1–12.1); T6: SG 29.8% (95% CI 28.7–30.8) versus ILM/OMM 14.2% (95% CI 13.1–15.3), difference 15.5 percentage points (95% CI 14.0–17.1); T9: SG 35.3% (95% CI 34.2–36.4) versus ILM/OMM 16.1% (95% CI 15.1–17.2), difference 19.2 percentage points (95% CI 17.7–20.7); and T12: SG 37.6% (95% CI 36.5–38.7) versus ILM/OMM 18.0% (95% CI 16.9–19.1), difference 19.6 percentage points (95% CI 18.1–21.1). The %TWL group‐by‐visit interaction was significant (Wald *χ* ^2^ = 894.15, df = 5, *p* < 0.001).

Repeated‐measures mixed‐effects analysis also showed a significant treatment‐by‐time interaction for BMI trajectory (Wald *χ* ^2^ = 178.59, df = 5, *p* < 0.001), indicating that BMI reduction over follow‐up differed significantly between SG and ILM/OMM.

#### 3.3.2 High Responders versus Low Responders

Conversely, male sex (OR 79.8, 95% CI 4.61–1380.52; *p* < 0.001), older age (OR 0.92 per year, 95% CI 0.86–0.98; *p* = 0.013), and lower baseline weight (OR 0.75 per kg, 95% CI 0.67–0.84; *p* < 0.0001) were associated with lower treatment response.

Treatment with SG was associated with lower odds of being a low responder compared with ILM/OMM (OR 0.04, 95% CI 0.001–0.67; *p* = 0.025).

Diabetes, cardiovascular disease, and dyslipidemia were not significantly associated with treatment response, although dyslipidemia showed a borderline association (OR 40.4, 95% CI 0.89–1832.7; *p* = 0.056) (Table S2).

**FIGURE 3:**
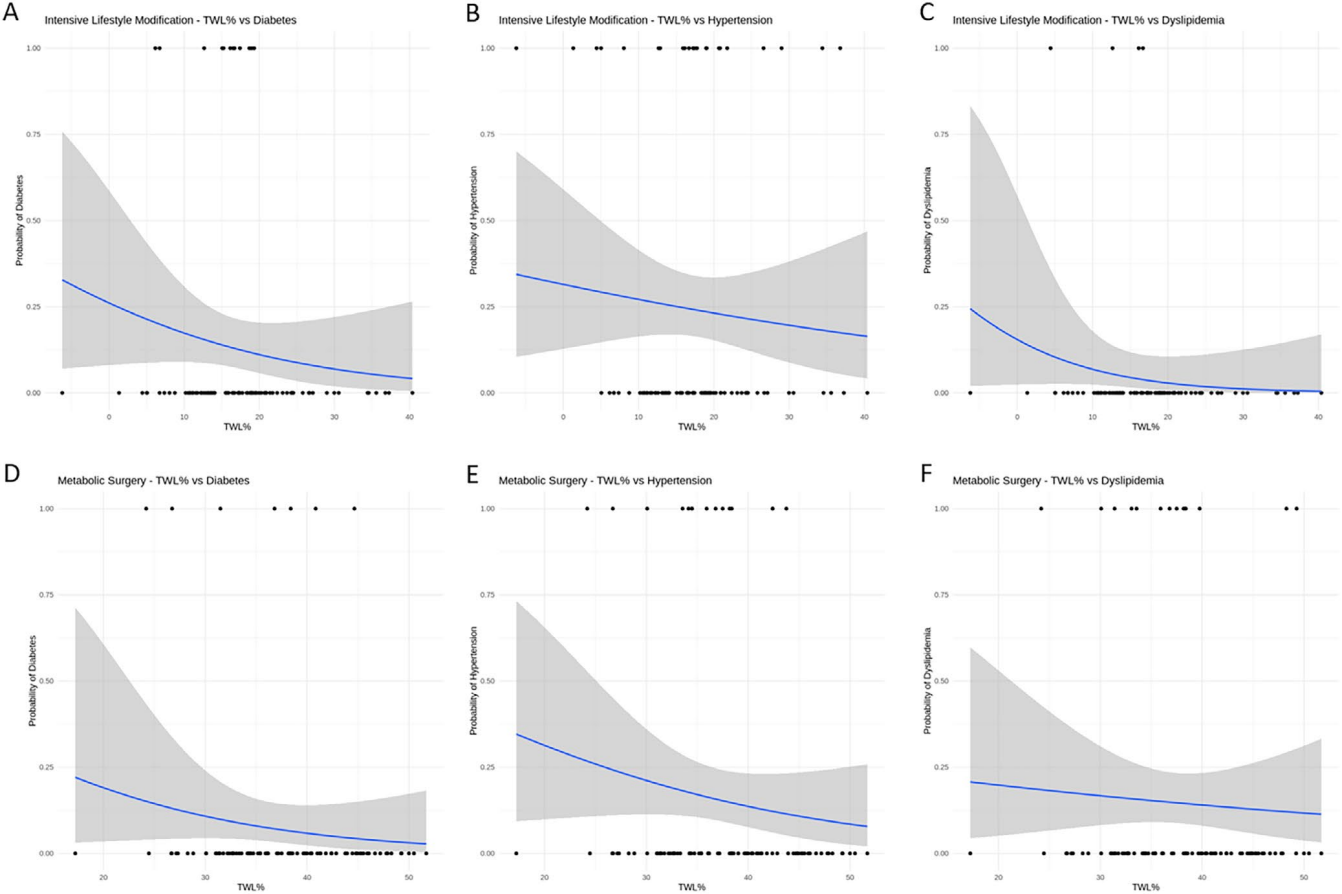
Relationship between %TWL and (A) diabetes, (B) hypertension, and (C) dyslipidemia in the ILM/OMM group. Relationship between %TWL and (D) diabetes, (E) hypertension, and (F) dyslipidemia in the SG group.

The probability curves for each condition as a function of %TWL are presented in Figure 3. The plots illustrate the lack of significant trends in the relationship between %TWL and the three conditions across both intervention groups.

### 3.4 Metabolic Outcomes

Hypertension, cardiovascular disease, dyslipidemia, type 2 diabetes and diabetes medication use were analysed as repeated binary outcomes. Observed *n* (%) values are retained in Table 2, and repeated‐measures logistic models provide predicted probabilities and between‐group differences with 95% confidence intervals. At T12, the model‐based predicted hypertension prevalence was 15.8% after SG versus 25.2% after ILM/OMM, corresponding to a difference of −9.4 percentage points (95% CI −20.8 to 2.0). Predicted dyslipidemia prevalence was 14.7% after SG versus 4.2% after ILM/OMM, corresponding to a difference of 10.6 percentage points (95% CI 2.4–18.8).

For T2D, the SG group showed a reduction in prevalence from 10.5% to 7.4%, while the ILM/OMM group maintained a constant prevalence of 12.6%, with no significant differences over time. Medication use for diabetes, including insulin, decreased slightly in the SG group, with 5.3% on insulin at T12 compared to 6.3% at baseline, while the ILM/OMM group saw no meaningful change (remaining at 1.1% on insulin).

A weight loss subgroup analysis in patients affected by T2D was performed and revealed that patients receiving Semaglutide achieved a mean weight loss of 15.0%, with the 25th, 50th, and 75th percentiles at 14.5%, 16.3%, and 17.7% respectively. This relatively narrow distribution suggests consistent, predictable outcomes with this GLP‐1 receptor agonist treatment.

In contrast, SG patients demonstrated substantially greater weight reduction, with a mean weight loss of 34.1%. The interquartile range for surgical patients was wider (25th percentile: 29.1%, 50th percentile: 36.8%, 75th percentile: 39.6%), indicating more variable but generally superior outcomes compared to pharmacotherapy.

### 3.5 Adverse Events and Postoperative Complications

In the ILM/OMM group the most commonly reported adverse events were gastrointestinal symptoms and included nausea in 16 (16.8%) patients, particularly during the initial 3 weeks of treatment. Diarrhoea was reported in 9 (9.5%) subjects, while vomiting affected 8 (8.4%) subjects, and constipation was experienced by 5 (5.3%). No drug discontinuation occurred in this group due to gastrointestinal symptoms. Two patients required dose reduction, with de‐escalation from 2.4 mg to 1.0 mg due to tolerability issues.

In the surgical group no minor or major complication was reported in the short‐term (30 days postoperatively). Fourteen (14.7%) patients complained of gastroesophageal reflux symptoms at a mean of 28 weeks after surgery. All patients had a resolution of symptoms after proton pump inhibitor prescription.

## 4 Discussion

SG was associated with greater and more rapid BMI reduction over a 1‐year follow‐up period compared with ILM/OMMs. In contrast, the medical strategy produced a more gradual weight‐loss trajectory while still providing clinically meaningful metabolic improvements over 1 year.

The primary outcome demonstrated a markedly greater reduction in BMI in the SG group (45.6 ± 8.1 to 28.3 ± 5.3 kg/m^2^) compared to the ILM/OMM group (45.3 ± 8.0 to 31.6 ± 4.2 kg/m^2^), (*p* < 0.01). This finding aligns with existing literature that establishes SG as a more effective intervention compared to medical treatment for substantial and sustained weight loss [10, 11, 12].

The surgical reduction in gastric volume and its subsequent impact on appetite regulation likely underpin these results. In contrast, ILM/OMM's reliance on patient adherence to dietary, pharmacological, and physical activity protocols may limit its efficacy, despite the demonstrated benefits of GLP‐1 receptor agonists like semaglutide [13]. GLP‐1 agonists and glucose‐dependent insulinotropic polypeptide/GLP‐1 receptor agonists can attain approximately 8% to 21% weight loss, and bariatric surgery can attain approximately 25%–30% weight loss [14].

The time‐to‐event analysis showed that patients undergoing SG reached ≥ 25% TWL earlier than those receiving ILM/OMMs. The median time to achieve this threshold was 6 months in the surgical group whereas the median was not reached by 12 months in the medical group, with a significant difference between groups (log‐rank *p* < 0.0001). In Cox regression analysis, SG was associated with a higher probability of achieving ≥ 25% TWL during follow‐up (HR 13.85, 95% CI 7.81–24.58). These findings reflect the more rapid weight reduction observed after metabolic surgery, whereas medical therapy was associated with a slower progression toward substantial weight loss. This study extends current evidence by moving beyond static weight‐loss outcomes to a trajectory‐based evaluation, incorporating time to achieving a clinically meaningful threshold and complete follow‐up in a real‐world matched cohort.

The degree of weight reduction observed with semaglutide in our study (~18% TWL at 12 months) is consistent with recent large‐scale trials such as STEP 1 and STEP 8, which reported average losses of 14.9%–17.4% [13, 15]. These results support semaglutide's robust efficacy as a pharmacological option, particularly for patients who are unwilling or ineligible for surgery. Our subgroup analysis of patients with T2D showed that semaglutide produced more uniform outcomes, whereas SG resulted in more variable but substantially greater weight loss. This observation is supported by recent registry data suggesting that while GLP‐1 receptor agonists yield consistent results across broad populations, surgery tends to produce higher but more heterogeneous responses [16]. Real‐world data suggest that fewer than 50% of patients remain on GLP‐1 receptor agonist therapy at 1 year, highlighting challenges in long‐term adherence [16, 17, 18].

The landmark STEP 1 trial showed mean weight loss of 14.9% versus 2.4% with placebo at 68 weeks in 1961 adults with obesity or overweight with comorbidities [13]. In the diabetic population studied in the STEP 2 trial, semaglutide achieved 9.6% mean weight loss compared to 3.4% with placebo, demonstrating efficacy even in metabolically complex patients while simultaneously improving glycemic control with HbA1c reductions of 1.6% versus 0.4% with placebo [19]. However, the STEP 4 withdrawal study revealed that discontinuation of semaglutide results in regain of approximately two‐thirds of lost weight over 48 weeks, emphasizing the chronic nature of obesity and the requirement for indefinite treatment to maintain benefits [20].

The impact on obesity‐related comorbidities in our cohort was modest within the first year. Improvements in hypertension and dyslipidemia were observed, with a trend toward greater benefit in the SG group, though statistical significance was limited, likely due to sample size and short follow‐up. T2D remission was greater after SG (from 10.5% to 7.4%), echoing prior studies [10, 11, 12], which demonstrated superior long‐term glycemic control after surgery compared to medical therapy. Semaglutide, while highly effective at lowering glucose levels in T2D patients, did not reduce prevalence in our study, perhaps reflecting differences in disease severity, baseline glycemic control, and relatively short follow‐up.

The SG group showed a greater relative improvement in hypertension prevalence (from 29.5% to 15.8%) than the ILM/OMM group (25.3%–23.2%), though these differences did not reach statistical significance. Improvements in dyslipidemia were more pronounced in the ILM/OMM group (from 6.3% to 4.2%) than in the SG group (15.8%–14.7%), potentially linked to the lipid‐modifying effects of pharmacological agents used and/or baseline differences between groups. These findings suggest that while SG drives weight loss more effectively, ILM/OMM may offer targeted benefits in lipid metabolism.

While bariatric surgery is highly effective, it is accompanied by certain risks, including surgical complications and the possibility of nutrient deficiencies that require ongoing monitoring throughout a patient's life [21, 22]. For individuals unable or unwilling to undergo surgery, intensive lifestyle modifications remain a viable alternative, particularly when combined with GLP‐1 receptor agonists. The observed lipid improvements in the ILM/OMM group suggest a potential role for these medications in managing dyslipidemia and lowering cardiovascular risk. However, the more modest weight loss outcomes underline the importance of setting realistic expectations for patients pursuing nonsurgical treatments.

The therapeutic landscape of obesity management is rapidly evolving, with an expanding shift toward less invasive and increasingly effective pharmacological options. However, these advances do not obviate the need for careful patient selection. Not all individuals derive equivalent benefit from medical therapy alone, particularly those with very high baseline BMI, in whom achieving and sustaining a clinically meaningful weight‐loss trajectory remains challenging. Long‐term considerations are equally critical. While bariatric surgery entails substantial upfront costs, accumulated evidence indicates that over a 10‐year horizon it is often more cost‐effective than continuous obesity management medications, which require potentially lifelong use [23]. Moreover, real‐world adherence to long‐term pharmacotherapy is frequently suboptimal, contributing to attenuated weight‐loss durability and less favourable long‐term outcomes compared with those reported in tightly controlled randomized trials [22]. These considerations emphasize the gap between outcomes observed in prospective randomized controlled trials with intensive follow‐up and those achieved in routine clinical practice. Moving forward, the most promising strategy lies not in a binary choice between surgery and medical therapy, but in integrated, personalized treatment pathways. The use of obesity management medications as a bridge to surgery, as adjuvant therapy to enhance or maintain surgical outcomes, or as part of combined and sequential approaches may optimize both efficacy and safety. Such tailored strategies are likely to better reflect real‐world needs and represent the future direction of comprehensive obesity care. Furthermore, the emergence of potent dual‐agonist drugs like tirzepatide and promising combinations such as CagriSema [24, 25] may soon challenge the traditional hierarchy of obesity treatment.

The present study captures early treatment responses over 1 year but does not address long‐term durability of weight loss. Evidence from randomized trials of GLP‐1 receptor agonists suggests that weight regain frequently occurs after treatment discontinuation, highlighting the importance of long‐term pharmacologic adherence. In contrast, bariatric surgery has demonstrated sustained weight loss and metabolic benefits over longer follow‐up periods in multiple longitudinal studies. Ongoing follow‐up of our cohort will allow future evaluation of long‐term maintenance of weight loss and metabolic outcomes. Although weight reduction was greater after surgery, improvements in metabolic comorbidities were observed in both treatment groups. These findings suggest that substantial metabolic benefits can be achieved through both surgical and intensive medical approaches, though the magnitude and timing of these effects may differ. The differing intervention structures, including the use of a very low‐calorie diet in the medical group and the absence of equivalent dietary restriction after surgery, introduce potential structural bias. Therefore, findings should be interpreted as reflecting integrated treatment strategies rather than isolated effects of individual components. Notably, the more intensive dietary and behavioural intervention in the ILM/OMM group would be expected to bias outcomes in favour of the medical strategy; however, the observed between‐group differences remained substantial.

The real‐world design of this study provides insight into clinical decision‐making outside randomized trials. Treatment selection reflected shared decision‐making between clinicians and patients, incorporating individual preferences, comorbidity profiles, and eligibility for surgery. As such, the results may better reflect routine clinical practice than highly controlled trial settings.

Finally, adverse events were limited and differed between groups. Gastrointestinal side effects in the semaglutide arm were frequent but manageable, consistent with reports from the STEP trials [13, 15, 19, 20]. In contrast, SG was not associated with short‐term surgical complications, though 14.7% of patients developed postoperative gastroesophageal reflux—an increasingly recognized long‐term risk after SG [26]. This study was not designed as a comprehensive comparative assessment of safety or quality of life outcomes, and these findings should be interpreted accordingly.

### 4.1 Strengths and Limitations

Strengths include the rigorous follow‐up and comprehensive evaluation of both anthropometric and metabolic outcomes. The prospective design, absence of loss to follow‐up, and evaluation of both weight‐loss trajectories and time to achieving ≥ 25% TWL strengthen the analysis. Given the real‐world design of the study, both lifestyle and pharmacological interventions were individualized, resulting in some degree of heterogeneity in treatment exposure. Several limitations should also be acknowledged. Given the non‐randomized design, causal inference cannot be established. Despite matching and propensity score adjustment, residual confounding cannot be excluded because of the observational design. Second, the 1‐year follow‐up, while informative, is insufficient to assess the long‐term durability of weight loss and metabolic benefits. Third, the relatively small sample size for some subgroups, such as patients with T2D, may have limited the power to detect differences in secondary metabolic outcomes. Detailed quantitative adherence and persistence metrics were not systematically collected, although no patient discontinued semaglutide during follow‐up. Additionally, measures of insulin resistance (e.g., HOMA‐IR), inflammatory biomarkers (e.g., hs‐CRP), and patient‐reported outcomes were not assessed.

## 5 Conclusions

Sleeve gastrectomy was associated with greater and more rapid weight loss than intensive medical therapy over 1 year, with earlier achievement of ≥ 25% total weight loss. Both approaches produced clinically meaningful metabolic improvements. These findings support individualized, patient‐centered treatment selection. As novel pharmacotherapies continue to evolve, further research should assess their long‐term comparative effectiveness and costs against bariatric surgery. Longer‐term studies are needed to evaluate durability and real‐world effectiveness.

## Author Contributions

All authors contributed to the study design, manuscript writing, data collection, and interpretation of the results. All authors provided critical feedback and helped shape the research, analysis, and manuscript. All authors have approved the final version of the article.

## Funding

The authors have nothing to report.

## Conflicts of Interest

The authors declare no conflicts of interest.

## Supporting information


**Table S1:** Baseline covariate balance before and after overlap weighting.

**Table S2:** Multivariate logistic regression analysis of factors associated with treatment response.

**Table S3:** Model diagnostics and fit indices for the continuous mixed‐effects models.

## Data Availability

Data is available upon reasonable request to the corresponding author.
