# Supplementary material for: Impact of Sleeve Gastrectomy Versus Intensive Lifestyle Modifications With Obesity Management Medications on BMI Trajectory and Target Attainment: A Prospective Matched Cohort Study
Source: Diabetes Obes Metab. 2026 May 31;28(8):7126–36. doi: 10.1111/dom.70912 (PMC13341333; doi:10.1111/dom.70912)
Supplement: Supplementary file 1 — Table S1: Baseline covariate balance before and after overlap weighting. [file DOM-28-7126-s003.docx]

| Variable | SG (n=95) Mean / % | ILM/OMM (n=95) Mean / % | SMD (unweighted) | SMD (overlap weighted) |
| --- | --- | --- | --- | --- |
| Age (years) | 42.6 | 48.8 | -0.506 | -0.007 |
| Female sex, n (%) | 83.2% | 69.5% | 0.324 | 0.009 |
| Body weight (kg) | 124.3 | 105.7 | 0.886 | 0.022 |
| BMI (kg/m²) | 45.6 | 45.3 | 0.035 | -0.012 |
| Diabetes mellitus, n (%) | 16.8% | 12.6% | 0.118 | -0.006 |
| Diabetes medications, n (%) | 17.9% | 9.5% | 0.246 | 0.002 |
| Metformin, n (%) | 16.8% | 8.4% | 0.254 | 0.003 |
| GLP-1 receptor agonist, n (%) | 0.0% | 6.3% | -0.365 | -0.082 |
| SGLT2 inhibitor, n (%) | 0.0% | 1.1% | -0.145 | -0.028 |
| DPP4 inhibitor, n (%) | 0.0% | 0.0% | N/A | N/A |
| Insulin, n (%) | 6.3% | 1.1% | 0.281 | 0.005 |
| Hypertension, n (%) | 33.7% | 25.3% | 0.185 | 0.001 |
| Dyslipidemia, n (%) | 17.9% | 6.3% | 0.359 | 0.009 |
| Cardiovascular disease, n (%) | 1.1% | 7.4% | -0.317 | -0.009 |

**Supplementary Table 1.** Baseline covariate balance before and after overlap weighting (obesity medication excluded as post-enrollment variable).

SMD = standardized mean difference. Values in SMD unweighted bold indicate |SMD| > 0.2 (meaningful imbalance). Values in SMD overlap weighted indicate |SMD| < 0.1 (good balance). Obesity medication was excluded from the propensity model as it was prescribed after enrollment. Overlap weighting was selected as it provided the best covariate balance (mean |SMD| = 0.015, max |SMD| = 0.082).
