# Supplementary material for: Impact of Sleeve Gastrectomy Versus Intensive Lifestyle Modifications With Obesity Management Medications on BMI Trajectory and Target Attainment: A Prospective Matched Cohort Study
Source: Diabetes Obes Metab. 2026 May 31;28(8):7126–36. doi: 10.1111/dom.70912 (PMC13341333; doi:10.1111/dom.70912)
Supplement: Supplementary file 2 — Table S2: Multivariate logistic regression analysis of factors associated with treatment response. [file DOM-28-7126-s002.docx]

| Variable | OR | 95% CI | P value |
| --- | --- | --- | --- |
| High response model |  |  |  |
| Female sex | 68.9 | 4.42–1072.98 | <0.001 |
| Baseline weight (per kg) | 1.16 | 1.07–1.26 | <0.0001 |
| Low response model |  |  |  |
| Male sex | 79.8 | 4.61–1380.52 | <0.001 |
| Age (per year) | 0.92 | 0.86–0.98 | 0.013 |
| Baseline weight (per kg) | 0.75 | 0.67–0.84 | <0.0001 |
| Sleeve gastrectomy (vs ILM/OMM) | 0.04 | 0.001–0.67 | 0.025 |
| Dyslipidemia | 40.4 | 0.89–1832.7 | 0.056 |

**Supplementary Table 2.** Multivariate logistic regression analysis of factors associated with treatment response. Values are derived from multivariate logistic regression models. Odds ratios (ORs) with 95% confidence intervals (CIs) are reported. Continuous variables are expressed per unit increase. (ILM/OMM: intensive lifestyle modification with obesity management medications).
