# Supplementary material for: Impact of Sleeve Gastrectomy Versus Intensive Lifestyle Modifications With Obesity Management Medications on BMI Trajectory and Target Attainment: A Prospective Matched Cohort Study
Source: Diabetes Obes Metab. 2026 May 31;28(8):7126–36. doi: 10.1111/dom.70912 (PMC13341333; doi:10.1111/dom.70912)
Supplement: Supplementary file 3 — Table S3: Model diagnostics and fit indices for the continuous mixed‐effects models. [file DOM-28-7126-s001.docx]

| Outcome | Observations | Patients | AIC | BIC | Random intercept variance | Residual variance | Group x visit chi-square | df | P value |
| --- | --- | --- | --- | --- | --- | --- | --- | --- | --- |
| Weight (kg) | 1137 | 190 | 7954.717 | 8025.223 | 273.262 | 32.286 | 876.964 | 5 | <0.001 |
| BMI (kg/m²) | 1137 | 190 | 6405.351 | 6475.857 | 24.888 | 10.075 | 178.589 | 5 | <0.001 |
| %TWL | 1137 | 190 | 6761.72 | 6832.226 | 11.893 | 16.539 | 894.152 | 5 | <0.001 |

**Supplementary Table 3.** Supplementary model diagnostics for continuous mixed-effects models
